# Transcriptome changes in undifferentiated Caco-2 cells exposed to food-grade titanium dioxide (E171): contribution of the nano- and micro- sized particles

**DOI:** 10.1038/s41598-019-54675-0

**Published:** 2019-12-04

**Authors:** Héloïse Proquin, Marloes C. M. Jonkhout, Marlon J. Jetten, Henk van Loveren, Theo M. de Kok, Jacob J. Briedé

**Affiliations:** 1 Department of Toxicogenomics, GROW institute of Oncology and Developmental Biology, Maastricht University, P.O. Box 616, 6200 MD Maastricht, the Netherlands; 2 Department of Cellular and Molecular Medicine, KU Leuven, Herestraat 49, box 901 3000 Leuven, Belgium; 3 Complex Tissue Regeneration (CTR), Institute for Technology-Inspired Regenerative Medicine (MERLN), Maastricht University, P.O. Box 616, 6200 MD Maastricht, the Netherlands

**Keywords:** Cancer genomics, Cancer genomics

## Abstract

The food additive titanium dioxide (TiO_2_), or E171, is a white food colorant. Recent studies showed after E171 ingestion a significantly increased number of colorectal tumours in a colorectal cancer mouse model as well as inflammatory responses and dysregulation of the immune system in the intestine of rats. In the mouse colon, E171 induced gene expression changes related to oxidative stress, impairment of the immune system, activation of signalling and cancer-related processes. E171 comprises nanoparticles (NPs) and microparticles (MPs). Previous *in vitro* studies showed that E171, NPs and MPs induced oxidative stress responses, DNA damage and micronuclei formation. This study aimed to investigate the relative contribution of the NPs and MPs to effects of E171 at the transcriptome level in undifferentiated Caco-2 cells by genome wide microarray analysis. The results showed that E171, NPs, and MPs induce gene expression changes related to signalling, inflammation, immune system, transport and cancer. At the pathway level, metabolism of proteins with the insulin processing pathway and haemostasis were specific to E171 exposure. The gene expression changes associated with the immune system and inflammation induced by E171, MPs, and NPs suggest the creation of a favourable environment for colon cancer development.

## Introduction

Titanium dioxide (TiO_2_) is widely used as a food colorant in, among others, sweets, cookies, cake icing, and salad dressings^1–3^. TiO_2_ has been approved as a food additive in 1969 by the European Union (EU) under the name of E171^4^. This approval was based on a report written by the Joint WHO/FAO expert committee of Food Additives (JECFA) referring to the results of 5 studies published in 1927, 1928, 1950, 1955 and 1963 and one unpublished study in 1962 assessing TiO_2_ absorption and toxicity in animals after ingestion^5^. Based on the results of these studies, JECFA concluded that TiO_2_ was free from toxic effects on account of its insolubility and inertness^6^. Therefore the EU classified E171 as a non-active compound and there is no limit of authorised concentration in food products. In the USA, the Food and Drug Administration (FDA) approved the use of TiO_2_ as a food additive in 1966 and TiO_2_ should not exceed 1% by total weight of the product^7^.

E171 is a mixture of different particles with sizes in both the nano- and the micro-range. Characterisation of E171 by our group showed that E171 comprises 39% of nanoparticles (NPs) and 61% of microparticles (MPs)^8^. These results are in line with the size distribution observed in commercially purchased E171 from different manufacturers and in E171 extracted from food products by Scanning Electron Microscopy (SEM) or Transmission Electron Microscopy (TEM)^1,9–11^. These results showed that, in E171, the NPs fraction comprised 25 to 40% of all material, the remainder being MPs.

More recently, a limited number of animal studies have been published that provide evidence on the potential adverse effects after ingestion of E171 (2 in mice and 2 in rats). The first described effects of E171 ingestion in a murine colorectal cancer model^12^. After 10 weeks of intragastric ingestion of 5 mg/kg bw/day of E171 in combination with the genotoxicant azoxymethane (AOM) and the irritant dextran sodium sulphate (DSS), the number of tumours in the colon was significantly increased as compared to the control (AOM/DSS). The second *in vivo* study aimed to investigate the effects of E171 on the immune system in rats^13^. Bettini *et al*. showed that, after intragastric exposure of 10 mg/kg bw/day of E171 for 7 days, a decrease of regulatory T and Th cells and an increase production of IFN-γ in Peyer’s patches were observed which would eventually lead to an impairment of the immune system. A third study applying a long term (100 days) exposure, in a chemically induced carcinogenesis rat model, showed that E171 (10 mg/kg bw/day) promoted colon carcinogenesis by inducing preneoplastic lesions as well as the growth of aberrant crypt foci and mucosal low-grade inflammation^13^. The fourth *in vivo* study was performed on a normal BALB/c mouse model in which intragastric ingestion by oral gavage of 5 mg/kg bw/day of E171 was executed for 2, 7, 14, and 21 days^14^. Gene expression changes were observed in the colon which indicated oxidative stress responses, an impairment of the immune system, activation of signalling, and cancer-related genes.

More studies have been performed after ingestion of the smallest fraction of TiO_2_, the NPs fraction, indicating the translocation of the TiO_2_ NPs to the blood stream and other organs. Several groups showed that after intragastric administration of TiO_2_ NPs, these particles were found in the brain, blood, liver, and kidneys^15–18^. In the gut, inflammatory response via cytokine production or NLRP3 inflammasome, and dysregulation of the innate and adaptive immune system were identified after ingestion of TiO_2_ NPs^13,19,20^.

Two *in vivo* studies have been performed on MPs to assess translocation and toxicity. After intragastric ingestion, the particles were found in spleen, brain, while an inflammatory response in the small intestine was observed detected by an increased inflammatory cytokine production and T CD4^+^ cell proliferation^16,20^.

Few *in vitro* studies show the potential of E171 as well as TiO_2_ NPs to induce oxidative stress in Caco-2, Caco-2/HT29-MTX, human epidermal, and human amnion epithelial (WISH) cells^9,21–23^. In the exact same cell lines, DNA damage was also observed after exposure to E171 and NPs of TiO_2_. These results are in line with the results observed by our group where oxygen radical formation was detected in Caco-2 cells by the MPs of TiO_2_ and by E171 and NPs of TiO_2_ in a cell-free environment. Additionally, induction of DNA damage and micronuclei was measured after exposure to E171, NPs, and MPs of TiO_2_ in Caco-2 cells and HCT116 respectively^8^. In addition, induction of micronuclei was observed in HCT116 cells^8^. Another *in vitro* study showed that E171 extracted from gum disrupted the normal arrangement of constituent microvilli of the Caco-2_BBe1_ brush border^24^.

The aim of our study was to investigate the relative contribution of the NPs and MPs fractions to the effects of E171 at the transcriptome level. This investigation was performed using *in vitro* exposure of undifferentiated Caco-2 cells to E171 as well as the NPs and MPs fractions of TiO_2_ and assessing genome wide gene expression analysis. We specifically performed this study on undifferentiated Caco-2 cells because it was shown that these cells better reflect a gene expression pattern of human colon cancer cells in comparison to differentiated Caco-2 cells^25^. The set-up of the study design and transcriptomics analysis was linked to data on undifferentiated Caco-2 cells regarding dose-dependent cytotoxicity, oxidative stress DNA damage^8^. Undifferentiated enterocytes also form a subpopulation of cells in the colon and a comparison showed that undifferentiated Caco-2 cells are more sensitive towards particle toxicity and are therefore a preferred target in this type of studies^21^. We assumed that because of its small size and higher surface area, the NPs fraction would show the most pronounced effect on gene expression. Therefore, we first performed a time course study (2, 4, and 24 h of exposure) with this fraction to identify the most relevant time point at which the highest number of genes and related biological processes would be influenced. Next, we exposed undifferentiated Caco-2 cells to E171, and the MPs fraction at the optimal duration (24 h) to establish the relative contribution of each fraction to the various gene expression changes and related biological processes.

## Results

### Time course responses to TiO_2_ NPs exposure

We first exposed undifferentiated Caco-2 cells to TiO_2_ NPs for 2, 4, and 24 h to identify what time point would show the most abundant and pronounced transcriptomic response.

### Differentially expressed genes

After LIMMA, DEG were identified per time point and shown in Table 1 including different fold changes (FC) cut-offs. Only with a FC ≥ 1.5 and a p-value < 0.05, a gradual increase of the number of DEG was observed. Furthermore, when using a less stringent FC like 1.2, noise might be introduced to the gene expression data while with a more stringent FC (1.8), part of the biological effect might be lost. Therefore, the value of 1.5 was used as a cut-off value for FC. Due to the absence of DEG when including an adjusted p-value, the analysis was further performed with a regular p-value of 0.05 and a FC ≥ 1.5. Under these conditions, 234 DEG were found after 2 h exposure, which slightly increased to 242 DEG after 4 h and to 281 DEG after 24 h.

**Table 1:** DEG per time point after Caco-2 cells exposure to TiO_2_ NPs.

	FC ≥ 1.2			FC ≥ 1.5			FC ≥ 1.8		
	2 h	4 h	24 h	2 h	4 h	24 h	2 h	4 h	24 h
|FC|	3827	5220	3916	806	797	623	253	155	248
Upregulated	2004	2051	1978	595	503	234	214	63	169
Downregulated	1823	3169	1938	211	294	389	39	92	79
p.val < 0.05	532	1991	470	532	470	1991	532	1991	470
adj.p.val < 0.05	0	3	0	0	0	3	0	3	0
**|FC| and p.val**	434	1532	432	**234**	**242**	**281**	121	88	129
|FC| and adj.p.val	0	3	0	0	0	1	0	1	0

A LIMMA test was used in R with a fold change (FC) of > = 1.2, 1.5 or 1.8 and a p-value < 0.05. DEG used for the analysis are shown in bold. |FC| = Fold Change, p.val* = *p-value, adj.p.val* = *adjusted p-value.

**Figure 1:**
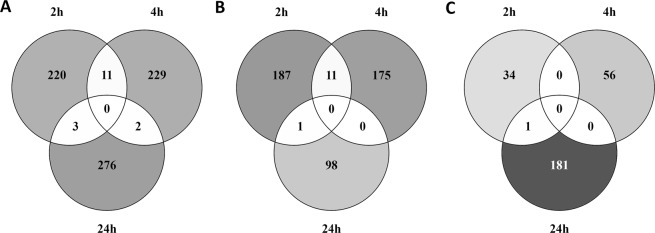
Overlap of DEG over time after exposure to TiO_2_ NPs for 2, 4 or 24 hours. Venn diagrams representing the overlap of (**A**) all DEG, (**B**) upregulated DEG and (**C**) downregulated DEG.

The overlap of DEG between the three time points was visualized using Venn diagrams, showing the overlap of all DEG (Fig. 1A), the overlap of upregulated DEG (Fig. 1B), and downregulated DEG (Fig. 1C) after exposure to TiO_2_ NPs for 2, 4, and 24 hours. Not a single gene was differentially expressed at all time points (Fig. 1A). There were 11 DEG overlapping between 2 and 4 h of exposure, all of which were upregulated (Fig. 1B). Two DEG were overlapping between 4 and 24 h of which 1 was upregulated and 1 downregulated (Fig. 1C) at both time points. Three DEG were overlapping between 2 and 24 h of exposure, none of these genes were expressed in the same direction at both time points. All the other DEG were time specific.

### Pathway and gene analyses

Using CPDB, the DEG for each time point were integrated in known pathways from different databases. The exposure to TiO_2_ NPs led to changes in gene expression that indicate different types of biological processes (Table 2). The result of this analysis is further described below.

**Table 2:** Pathway over-representation analysis (ORA) of the DEG.

Time of exposure	Biological function	Name pathway	p-value
2 h	Signal Transduction	Olfactory receptor activity	3.03E-09
		Olfactory transduction	4.73E-09
		Olfactory Signalling Pathway	8.72E-09
		GPCR downstream Signalling	6.47E-07
		Signalling by GPCR	1.60E-06
		GPCRs, Class A Rhodopsin-like	0.00518
		Signal Transduction	0.0125
	Immune system	*Staphylococcus aureus* infection	0.0113
		Dectin-2 family	0.0125
	Metabolism	Vitamin A and Carotenoid Metabolism	0.0297
		Retinol metabolism	0.0468
	Transport	Cation-coupled Chloride cotransporters	0.0125
4 h	Signal Transduction	Olfactory Signalling Pathway	1.57E-05
		Olfactory receptor activity	9.53E-05
		Olfactory transduction	9.53E-05
		GPCR downstream Signalling	0.000714
		Signalling by GPCR	0.000807
		GPCR Signalling-G alpha i	0.0223
		GPCR Signalling-pertussis toxin	0.0223
		VEGF binds to VEGFR leading to receptor dimerization	0.0223
		VEGF ligand-receptor interactions	0.0223
		GPCR Signalling-cholera toxin	0.0234
		GPCR Signalling-G alpha s Epac and ERK	0.0234
		GPCR Signalling-G alpha q	0.0234
		VEGF and VEGFR Signalling network	0.0234
		Signal Transduction	0.0234
		GPCR Signalling-G alpha s PKA and ERK	0.0234
	Metabolism	Serotonin and melatonin biosynthesis	0.0127
		Bile acid and bile salt metabolism	0.029
		Peptide hormone metabolism	0.029
		Synthesis of bile acids and bile salts via 24-hydroxycholesterol	0.029
		Biogenic Amine Synthesis	0.037
		Metabolism of Angiotensinogen to Angiotensins	0.0389
		Primary bile acid biosynthesis	0.0407
		Amine-derived hormones	0.044
	Transport	Ligand-gated ion channel transport	0.00701
		Transport of organic anions	0.0234
		Transmembrane transport of small molecules	0.029
		Transport of glucose and other sugars, bile salts and organic acids, metal ions and amine compounds	0.0389
	Neuronal system	Serotonin and anxiety	0.029
		Serotonergic synapse	0.0407
		Neuroactive ligand-receptor interaction	0.0446
24 h	Signal Transduction	Gastrin-CREB Signal Transduction pathway via PKC and MAPK	0.108
		Peptide ligand-binding receptors	0.108
		Class A/1 (Rhodopsin-like receptors)	0.108
		G alpha (i) Signal Transduction events	0.128
	Immune system	Negative regulation of MAPK pathway	0.131
	Metabolism	Glucocorticoid Receptor Pathway	0.00221
		Glycine, serine, alanine and threonine metabolism	0.0207
		Creatine metabolism	0.108
		Prostaglandin Synthesis and Regulation	0.108
		Metabolism of polyamines	0.128
	Transport	Bile salt and organic anion SLC transporters	0.128
	Neuronal system	TarBasePathway	0.128
		HCN channels	0.03
	Cancer	Photodynamic therapy-induced HIF-1 survival Signalling	0.128
	Gene expression (Transcription)	TP53 Regulates Transcription of Cell Cycle Genes	0.128
	Developmental biology	Endoderm Differentiation	0.128
		Developmental Biology	0.108
	Haemostasis	Hemostasis	0.0382

Pathways related to the DEG after ORA with ConsensusPathDB. The pathways were grouped per time points and per biological function.

After 2 h of exposure to TiO_2_ NPs, ORA showed that the modulated DEG were involved in pathways related to signal transduction with 7 pathways involved in olfactory, G protein-coupled receptor (GPCR) and other signal transduction. In addition, 2 pathways were associated with the innate immune system containing genes coding for C-type lectin domain, Fc fragment, HLA, and complement system. Two other pathways, vitamin A and retinol metabolism were affected after 2 h exposure. These 2 pathways were also implicated in increasing oxidative stress^26^. DEG after 2 h exposure were also involved in transport of molecules with HTR3B, GABRG2, and 2 solute carrier genes.

After 4 h of exposure to TiO_2_ NPs, 30 pathways associated to the DEG were observed after ORA. Different biological processes were identified such as signal transduction with 15 pathways involved in olfactory, GPCR, and VEGF signalling and metabolism with 8 pathways related to serotonin, bile acid, peptide hormones, and angiotensin metabolism. In addition, neuronal system was observed with 3 pathways associated to serotonin activity and neuroactive ligand-receptor interaction. Transport of small molecules was a biological function observed after 4 h exposure and contained 4 pathways involved in ligand-gated ion channel transport, transport of organic anion, and transport of glucose and other sugars, bile salts and organic acids, metal ions and amine compounds.

After 24 h exposure, ORA identified 18 pathways affected after exposure to TiO_2_ NPs. These pathways were involved in a various number of biological processes such as signal transduction with 4 pathways related to gastrin-CREB, peptide ligand binding, and GPCR signalling. Metabolism was also observed with 5 pathways including 2 pathways (creatinine and prostaglandin metabolism) involved in a response to oxidative stress^27,28^. Neuronal system biological function was also detected with 2 pathways containing genes involved in HCN channels. In addition, 2 pathways were observed in developmental biology including endoderm differentiation, 1 in immune system: negative regulation of MAPK pathway, 1 in cancer: photodynamic therapy-induced HIF-1 survival signalling, 1 in haemostasis, 1 in gene expression (transcription): TP53 regulates transcription of cell cycle genes, and 1 in transport of small molecules: bile salt and organic anion SLC transporters.

### Comparison of the different time points

No pathways after ORA were common to all time points but 7 were overlapping between 2 and 4 h of exposure to TiO_2_ NPs and were all related to signal transduction and transport of small molecules (Table 2). No affected pathways were overlapping between 4 and 24 h and between 2 and 24 h of exposure. Pathways classified according to their biological function showed that signal transduction, metabolism and transport of small molecules were common to all time points. In addition, between 2 and 24 h molecular changes were observed in the immune system.

**Figure 2:**
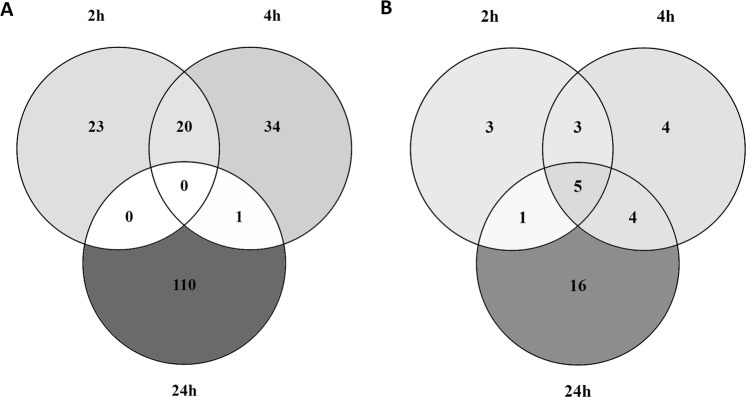
Overlap of GO terms and their biological processes over time. Venn diagram representing (**A**): the number of GO terms in common between 2, 4, and 24 h exposure to TiO_2_ NPs, and (**B**): the associated biological processes to the GO terms in common between 2, 4, and 24 h exposure to TiO_2_ NPs.

The same affected biological processes were observed when the GO terms associated to the DEG were analysed. Over time, the number of GO terms observed increased from 43 GO terms after 2 h of exposure, to 54 GO terms after 4 h and 111 GO terms after 24 h (Fig. 2A). Overlap was observed between 2 and 4 h with 20 GO terms involved in cellular response to stimulus, membrane, nervous system, receptor activity, signal transduction, and system process. Only one GO term was overlapping between 4 and 24 h, regulation of secretion. However, when the GO terms were classified according to their biological function, more overlap was observed. Figure 2B shows the different biological processes per time point with 12 after 2 h exposure, 12 after 4 h and 26 after 24 h. Of these 26 biological processes after 24 h exposure, 5 were in common with 2 and 4 h: cellular response to stimulus, nervous system, membrane, metabolism, immune system, 4 GO terms between 4 and 24 h: transport of small molecules, response to stress, secretion, and response to stimulus, and 1 GO term between 2 and 24 h: reproduction.

The results show the most abundant and diverse gene expression and pathway changes after 24 h exposure, for which reason we choose this time point to establish gene expression responses following MPs and E171 exposure.

### Comparison of transcriptome responses to different sizes of TiO_2_

#### Differentially expressed genes

In order to compare the gene expression responses induced by different fractions of E171, undifferentiated Caco-2 cells were also exposed to E171 and MPs for 24 h. Table 3 shows the number of DEG identified after LIMMA with the same FC and p-value cut-offs used for the time course with TiO_2_ NPs. After exposure to E171, the number of DEG was 3 times larger than after MPs and NPs exposure. As explained in the Materials and Methods, a new pre-processing was done on NPs with its time-matched control.

**Table 3:** DEG after exposure of Caco-2 cells to different TiO_2_ particles for 24 h.

	NPs	MPs	E171
|FC| > = 1.5	415	365	1040
Upregulated	148	136	63
Downregulated	267	229	977
p.val < 0.05	664	459	1198
adj.p.val < 0.05	0	0	0
**|FC| and p.val**	**121**	**121**	**365**
|FC| and adj.p.val	0	0	0

A LIMMA test was used in R with a fold change (FC) of >=1.5 and a p-value < 0.05 after *in vitro* exposure to E171, MPs, and NPs of TiO_2_. The number of DEG used for further analysis is shown in bold. |FC| = Fold Change, p.val = p-value, adj.p.val = adjusted p-value.

**Figure 3:**
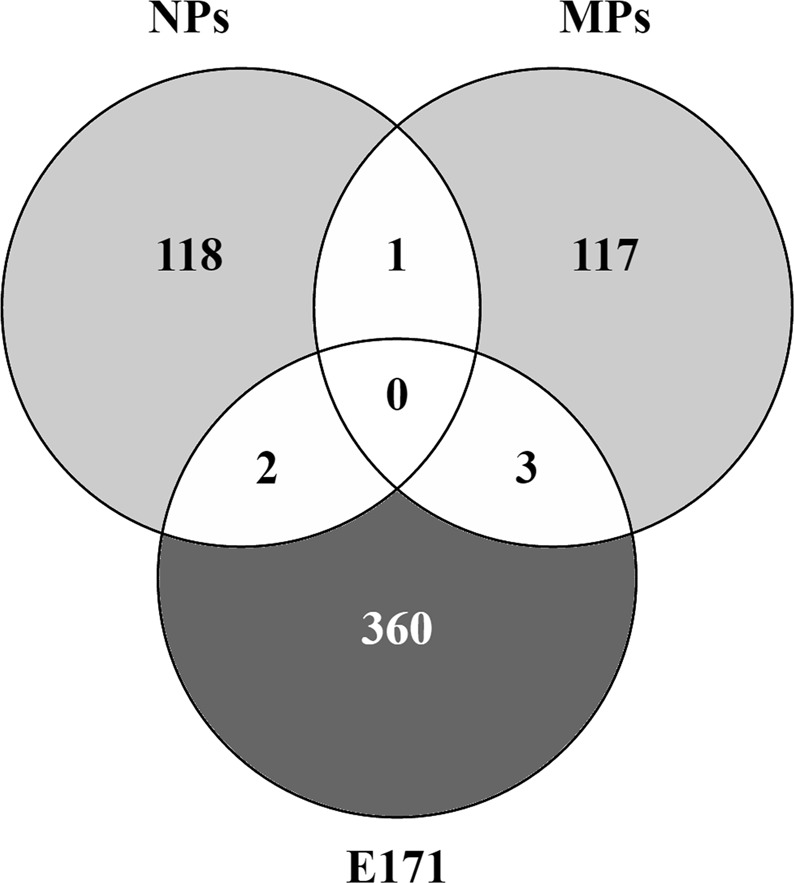
Unique and common DEG between E171 and the different size fractions of TiO_2_. Venn diagram showing the overlap of DEG after exposure of undifferentiated Caco-2 cells to E171, MPs, and NPs for 24 h.

Figure 3 shows that the DEG after exposure to the different sizes of TiO_2_ were mostly specific to each fraction, only a few genes were in common. One DEG was in common after MPs and NPs exposure: LGALS14, a galectin gene. Two DEG were in common between the NPs and E171 exposure: TCP11X2, a T-complex family gene and KCCAT333, a renal clear cell carcinoma-associated transcript but the exact function of these 2 genes remains unknown. When comparing the DEG after MPs and E171 exposure, 3 DEG were observed: WDR78, LOC401478, and ANKRD26P3. WDR78 gene is shown to be a target of the hedgehog activity. LOC401478 and ANKRD26P3 have unknown functions.

### Pathway and gene analyses

#### E171 exposure

ORA showed that after E171 exposure, 9 pathways involved in signal transduction were observed; 4 of them were related to GPCR, 3 to olfactory signalling, 1 to signal transduction and 1 to bone morphogenic protein signalling (Table 4). In addition, gene expression was affected in one pathway involved in haemostasis (endothelins), one in neuronal system (dopaminergic neurogenesis), one in metabolism of proteins (insulin processing), and another one in transport of small molecules (zinc efflux by solute carriers family).

**Table 4:** Pathways associated with the DEG after NPs, MPs and E171 exposure.

Time of exposure	Biological function	Name pathway	p-value
E171	Signal Transduction	GPCR downstream signalling	1.09E-08
		Signalling by GPCR	3.75E-07
		Olfactory Signalling Pathway	2.05E-06
		Olfactory transduction - Homo sapiens (human)	3.36E-05
		Olfactory receptor activity	6.69E-05
		GPCR ligand binding	0.000236
		Signal Transduction	0.000461
		G alpha (q) signalling events	0.00222
		Bone Morphogenic Protein (BMP) Signalling and Regulation	0.00845
	Transport of small molecules	Zinc efflux and compartmentalization by the SLC30 family	0.00406
	Neuronal system	Dopaminergic Neurogenesis	0.0004
	Developmental Biology	Interaction between L1 and Ankyrins	0.0041
	Haemostasis	Endothelins	0.00989
	Metabolism of proteins	Insulin processing	0.00406
MPs	Signal Transduction	Olfactory Signalling Pathway	0.0579
		Olfactory transduction - Homo sapiens (human)	0.0579
	Developmental Biology	CDO in myogenesis	0.0654
		Myogenesis	0.0654
		Netrin-1 signalling	0.0775
NPs	Metabolism	Glycine, serine, alanine and threonine metabolism	0.000543
		Creatine metabolism	0.000789
		Urea cycle and metabolism of amino groups	0.00514
		Glucocorticoid Receptor Pathway	0.00541
		Prostaglandin Synthesis and Regulation	0.00861
	Neuronal system	HCN channels	8.60E-05
		TarBasePathway	0.00126
		Potassium Channels	0.00689
	Transport of small molecules	Amino acid transport across the plasma membrane	0.00926

ORA analysis performed with CPDB on all the DEG after NPs, MPs and E171 exposure *in vitro* for 24 h.

In addition, gene analysis showed biological effects on the immune system with 34 DEG including BCL10, CD274, COLEC10, defensin, and IFI27 genes. Gene expression in metabolism was also influenced by E171 exposure with a total of 22 DEG including genes involved in ATP binding, cytochrome P450, bile acid, and prostaglandin. With 13 DEG involved in cholinergic receptor, calcium channels, potassium channels, and glutamate receptor, neuronal system was also affected by E171 exposure. The gene expression (transcription) biological function was altered by 12 DEG such as MTERF1 (mitochondrial transcription), SMURF1 (ubiquitin related gene), HIST1H3I (histone cluster), zinc finger genes, and TP53I11 (tumour protein p53 inducible protein 11). With 10 DEG each, 2 biological processes were modulated, cellular responses to external stimuli with genes coding for prostaglandin protein, histone cluster, and heat shock protein and disease with NAG20, NRG1, SLA2, CSNK1A1L (signalling by WNT in cancer). E171 exposure also affected, with less than 10 DEG each, gene expression in other biological processes such as metabolism of RNA, DNA repair, cell cycle, muscle contraction, extracellular matrix organization, organelle biogenesis and maintenance, cell-cell communication, chromatin organization, circadian clock, DNA replication, programmed cell death, and reproduction.

### MPs exposure

ORA showed that after MPs exposure, signal transduction was affected by the modulation of gene expression in 2 olfactory pathways. In addition, pathways involved in developmental biology were observed, mostly involved in the myogenesis (Table 4).

Gene analysis showed that after exposure to MPs for 24 h, gene expression was also affected in the immune system with defensins, interleukins, THRIL, BTC, TIMP4, and MPEG1 genes. In addition, 8 genes were related to transport of small molecules and vesicle-mediated transport (solute carriers, ABCG4, CLIC2, KIF25, MYH13 and LCN9). One DEG, up-regulated after MPs exposure, was found to be up-regulated in presence of oxidative stress (UCP1)^29^. Cancer development was observed by the modulation of genes involved in cell death (HIST1H1T, CASP14, and DNAJC3), cancer related genes (WNT8A and CASC8), gene expression (transcription) (ZNF570 and ZFP42), and metabolism of proteins (CALB2 and KLHDC3). Three DEG were found to be related to the extracellular matrix organisation (COL17A1, ARC, and TIMP4). Six others were modulated in the developmental biology biological function (CDH4, KRT14, POU3F3, NTN3, RGMA, and HFE2), 3 DEG in metabolism (ARG1, TPH2, and PNPLA2), and one DEG in neuronal system (CHRNA3).

### NPs exposure

As shown in Table 3, the new pre-processing was statistically more stringent. More than 90% of the DEG of the pre-processing of 24 h only was also present in the time course analysis. A pathway and gene analysis were performed with the 121 DEG of the new pre-processing and compared to the one performed with the 281 DEG of the time course analysis. The biological response after NPs exposure after 24 h exposure was similar to the 24 h time point described in the time course with pathways involved in HCN channels, transport of small molecules, and 5 metabolism pathways including creatinine and prostaglandin also involved in response to oxidative stress response (Table 4).

### Common and specific biological reactions

**Figure 4:**
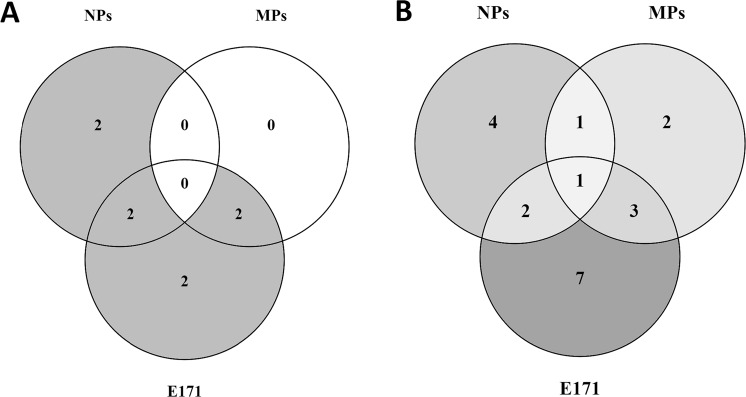
Overlap of the biological processes between the different sizes of TiO_2_. Venn diagram showing after exposure of undifferentiated Caco-2 cells to E171, MPs, and NPs for 24 h (**A**): the overlap of biological processes after ORA, (**B**): the overlap of the biological processes related to the GO terms.

Pathways extracted after ORA showed no common response between all the particles, all pathways were specific to each fraction. However, Fig. 4A shows that, after E171 and NPs exposure, 2 biological processes were in common: transport of molecules and neuronal system. After E171 and MPs exposure, signal transduction and developmental biology were common processes affected by gene expression changes. Both signal transduction pathways are related to the olfactory transduction.

Specific biological processes related to the DEG were also observed after NPs exposure: metabolism and response to stress. All biological processes after MPs exposure were common to E171 exposure. After E171 exposure, 2 specific biological reactions were observed: haemostasis and metabolism of proteins.

Figure 4B shows that one biological process extracted from the GO terms was common between the different types of TiO_2_, which is cellular response to stimuli. Furthermore, after E171 and NPs exposure gene expression was affected in 2 common biological processes: Transport and Membrane. Other common effects on gene expression were identified after E171 and MPs exposure in signal transduction, system processes, and neuronal system. After MPs and NPs exposure DEG were observed in the Binding biological process.

After NPs exposure, specific biological processes extracted from the GO terms of the DEG were related to metabolism, haemostasis, oxidative stress, and regulation of biological process. Two specific biological processes were observed after MPs exposure: reproduction and extracellular matrix organisation. The number of biological processes observed after E171 exposure was increased compared to NPs and MPs with receptor activity, immune system, DNA repair, muscle adaptation, catalytic activity, organ development, and transcription factor.

## Discussion

The aim of this study was to assess the gene expression changes in colonic cells after *in vitro* exposure to E171 and the contribution of the NPs and MPs fractions to these changes based on transcriptome analysis in undifferentiated Caco-2 cells. A transcriptomics study confirmed that in comparison to differentiated Caco-2 cells, undifferentiated Caco-2 cells better reflect a gene expression pattern of human colon cancer cells^25^. So this cell model shows a higher compatibility with our previous study in a mouse model of colon cancer that showed that E171 increased the number of tumours in the colon specifically^12^.

Although the majority of the DEG were not common between time points, this does not imply that the biological responses observed at earlier time points are transient. Interpretation of the biological functions of the genes using e.g. ORA and GO terms identified immune response, signal transduction and cancer signalling to be modulated after 2, 4, and 24 h. These dynamic biological responses are involved in consecutive mechanisms towards an increased risk of colon cancer development. First the optimal time point for the evaluation of gene expression responses was established to be 24 hours because this time point showed the highest number of DEG and affected pathways. Subsequently, cells were also exposed for 24 hours to MPs and E171.

A previous study performed by our group showed that TiO_2_ NPs induced ROS formation only in a cell-free environment^8^. In the current study, at the gene level, oxidative stress and related responses were observed: After 4 h the up-regulation of a protein coding gene (GML) involved in cellular response to stress and heat shock response and after 24 h with 2 DEG (TUBB2B and SCML4) involved in cellular response to stress, oxidative stress induced senescence and in a heat shock protein (HSP90) chaperone cycle. These results confirm previous studies which showed the capacity of TiO_2_ NPs to induce significant levels of oxidative stress in a cell-free environment as well as *in vitro* in Caco-2 and in human amnion epithelial (WISH) cells^9,21–23^. Oxidative stress is one of the factors that can induce DNA damage. Previously, we reported that TiO_2_ NPs induced significant levels of DNA damage after exposure to TiO_2_ NPs in undifferentiated Caco-2 cells as well as induction of micronuclei in HCT116 cells^8^. DNA damage was also shown by other studies performed in Caco-2, Caco-2/HT29-MTX co-culture system, and WISH cells after exposure to TiO_2_ NPs^9,21–23^. Here we show that, after 4 h exposure, 4 DEG (HAP1, GML, SPIDR, and REM1) were significantly expressed that are involved in DNA damage and repair. Two DEG were involved in the mechanisms of DNA repair activity either via a p53-target gene (GML)^30^ or via homologous recombination (SPIDR). Furthermore, HAP1 is known to be activated after DNA damage^31^ and REM1 expression is also shown to be modulated in CRC samples^32^.

Exposure to TiO_2_ NPs also induced gene expression changes related to the immune system. These changes start to manifest after 2 h via modulation of the expression of C4B and C3AR1 genes from the complement system as well as HLA-DRB5 gene involved in TCR signalling. After 4 h, no pathways involved in immune system were found, however, at the GO term level, cytokine production and tumour necrosis factor were observed. After 24 h exposure to TiO_2_ NPs, similar effects to 2 and 4 h exposure on the immune system were observed like on the innate immune system. In addition, specific gene expression changes related to IL-1 production, leukocyte migration, and chemokine signalling were observed, suggesting an impact on the innate and adaptive immune system. Overall 24 h exposure was the most pronounced time point regarding effects on the immune system because the majority of the 2 and 4 h exposure effects were found, including specific 24 h effects. TiO_2_ NPs exposure *in vivo* demonstrated impact on the immune response via a potent Th1/Th17 immune response after exposure to 10 mg/kg bw/day in rats of P25 TiO_2_^13^. Our results show that the induction of the immune response by TiO_2_ NPs might not only be induced via immune related organs, as seen *in vivo*, but also as a consequence of direct exposure of intestinal epithelium cells.

Furthermore, transport of small molecules and vesicle-mediated transport were observed by gene expression changes of solute carrier genes from 2 h to 4 h and 24 h exposure with 3, 7 and 7 DEG respectively. In addition, 2 cadherin genes, which play a role with cargo proteins in clathrin-independent endocytosis^33^, differentially expressed (4 h: CDH18, 24 h: PCDH9). Induction of genes in transport pathways may be explained by the small size and high surface area of TiO_2_ NPs. Previous experiments showed that TiO_2_ NPs can enter the cells as well as the nucleus^22,23,34^ and suggest that solute carriers and endocytosis might be two mechanisms by which the particles enter the cells^34–36^.

Overall, Fig. 2 shows that after 24 h exposure to TiO_2_ NPs, the gene expression changes were partly similar to other time points but also more diverse. Therefore, 24 h exposure was chosen to be the optimal time of exposure for the comparison of the different particles of TiO_2_. Effects of MPs and E171 at the molecular levels as well as the contribution of each fraction to these effects will be described below.

Like for the NPs, we have shown that MPs also induce oxidative stress after 1 h exposure in undifferentiated Caco-2 cells^8^. We found a significant up-regulation of UCP1, a gene with a very strong link to mitochondrial oxidative stress and DNAJC3, related to endoplasmic reticulum stress. UCP encodes for uncoupling proteins which limits ROS production in the mitochondrial respiratory chain after stimulation by O_2_^.^^−^^37,38^. DNAJC3 encodes for a protein involved in the unfolded protein response during endoplasmic reticulum stress^39^. Oxidative stress and cellular stress observed after MPs exposure might explain the induction of DNA damage and micronuclei previously observed after exposure of undifferentiated Caco-2 cells^8^. Here, gene expression change of one histone gene (HIST1H1T) was observed, which is involved in DNA repair^40,41^. In addition, after exposure to TiO_2_ MPs, DEG related to cancer, cell death, and gene transcription were observed like WNT8A and CASC8 which are highly correlated with CRC development and progression^42,43^. Furthermore, CASP14, a potent pro-apoptotic gene but also potentially involved in cell differentiation^44^ and RD3 a down-regulated gene normally expressed in the colon, while loss is associated with the development of neuroblastoma and other cancers^45^, were observed. So TiO_2_ MPs induced gene expression changes seem to indicate molecular responses that could reflect facilitation of cancer development.

Like exposure to TiO_2_ NPs, MPs also affected gene expression changes in relation to the immune system with the down-regulation of pro-inflammatory cytokine receptor IL-6R involved in the MAPK1 and MAPK3 activation and also in response to oxidative stress^46^. Other genes involved in the innate immune system such as defensins, interleukins, THRIL, BTC, TIMP4, and MPEG1 were additionally affected. TiO_2_ MPs ingested in the diet via E171 induced a pro-inflammatory and immune response which could increase the symptoms of inflammatory bowel disease via the NLRP3 inflammasome^47^ or when MPs are bound to Pathogen Associated Molecular Patterns (PAMPs) in the gastrointestinal lumen^48^.

Seven DEG were observed involved in transport of small molecules and vesicle-mediated transport. This included ABCG4, coding for a protein in the ATP-binding cassette (ABC) transporter superfamily which transport various molecules across extra- and intra-cellular membranes, and LCN9,involved in the transport of sugars, bile salts and organic acids, metal ions and amine compounds and were down- and up-regulated respectively. This suggests a misbalance of the efflux pumps which, correlated to induction of oxidative stress, may lead to accumulation of O_2_^.−^ ^49^.

After exposure to E171, the modulation of 2 genes (PTGES3 and HIST1H3I) related to cellular response to stress, oxidative stress induced senescence and cellular response to heat stress was observed. E171 additionally induced gene expression changes towards the development of inflammation which can also lead to DNA damage. As described for NPs and MPs, E171 also induced DNA damage in undifferentiated Caco-2 cells as well as induction of micronuclei in HCT116 cells^8^. Here, 4 DEG (POLB, NEIL3, POLD2 and LIG4) involved in DNA repair were modulated, like POLB involved in base excision and repair and POLD2 in DNA replication and repair. NEIL3 encodes for DNA glycosylases which cleaves bases damaged by ROS^50^. The protein coded by LIG4 is essential in DNA double-strand break repair through non-homologous end joining. Cancer related genes, which can be modulated as a consequence of DNA damage, were also observed, like down-regulation of the tumour suppressor CELF2^51^. In addition, NRG1 is involved in constitutive signalling by aberrant PI3K in cancer, CSNK1A1L in signalling by WNT in cancer, and MUCL1 in defective GALNT12 causes colorectal cancer.

E171 resulted in 31 DEG associated with the innate and adaptive immune system. These effects of E171 are in line with previous *in vivo* studies in which impairment of the immune system was observed such as that after ingestion of 5 mg/kg bw/day of E171 for 2, 7, 14 and 21 days, based on gene expression, BALB/c mice showed an impairment of the immune system^14^. One immune-related gene was commonly down-regulated in both *in vitro* and in the *in vivo* experiment, BCL10. It is involved in different processes in the immune system but also in protein ubiquitination and haemostasis. Another study described an impairment of the immune system via a Th1/Th7 response after exposure to 10 mg/kg bw/day in rats for 7 and 100 days^13^. The dysregulation of the immune system is a known factor that can lead to the development of cancer.

As described, several biological processes observed after E171 exposure were commonly induced by the MPs and NPs fraction such as gene expression changes in oxidative stress, DNA repair, immune system, and transport. Furthermore, other common effects are associated to signal transduction. In regards to the oxidative stress and DNA damage, the current results confirm the results previously observed via Electron Spin Resonance and the Comet assay in undifferentiated Caco-2 cells^8^.

In addition to common effects in gene expression in the immune system with the 3 types of TiO_2_, specific effects of NPs and E171 were observed in TLR cascade, advanced glycolysation, the MHC class I antigen processing and presentation and MHC class II presentation, and cytokine signalling with IL-1, IL-17, and IL-20.

In addition, 3 DEG involved in potassium channels were modulated. Changes in potassium channel expression are associated with colon carcinogenesis, however, it is not clearly defined whether those changes are inducing the development of cancer or a consequence reflecting dedifferentiation and ongoing proliferation^52^.

GO term analysis showed that Membrane was also observed as a common biological process after the NPs and E171 exposure. Within this biological process, 1 DEG after NPs and 1 DEG after E171 exposure were both coding for a late cornified envelope protein and both down-regulated. The cornified envelope is a structure which is responsible for the major barrier function of the skin^53^. Whether it plays a role in other epithelia barrier functions such as in the intestines is unknown. A dysregulation of the genes coding for these proteins might have a consequence on the integrity of the barrier in the gut and allow particles to pass through.

Exposure to NPs and E171 also induced gene expression changes in the cell cycle. The up-regulation of TUBB1 after NPs exposure involved in G2-G2/M and M phases and the down-regulation of HIST1H3I after E171 exposure also involved in M phase show a potential incidence of the TiO_2_ exposure on the regulation of cell cycle. Effects of titanium dioxide on the G2/M phase were already observed by Wu *et al*. who showed that anatase TiO_2_ NPs (25, 50, 100, and 200 μg/ml) in neuronal cells induced p53 and JNK activation in G2/M cell cycle arrest and apoptosis^54^.

After E171 and MPs exposure, specific biological processes and 3 DEG were commonly observed. Two out of three DEG have unknown function but WDR78 gene is shown to be a target of the Hedgehog activity and a promotor of FOXJ1 activity in zebrafish^55^. FOXJ1 is involved in the production of the motile cilia. In relation to the immune system, MPs and E171 affected gene expression in relation with the α-defensins including DEFA6 after MPs exposure and DEFA4 after E171 exposure were up- and down-regulated respectively. Normally present in epithelial cells, the defensins are antimicrobial peptides contributing to the mucosal host defence by the action of permeabilization of the microbe membrane^56^. Gene expression changes after MPs and E171 exposure were associated with signalling, notably olfactory signalling.

After MPs exposure, 2 DEG involved in cell junction organisation were modulated and 1 DEG after E171 exposure. More precisely 2 DEG (E171: CDH18 and MPs: CDH4) were both coding for cadherin surface proteins. These proteins not only play an important role in endocytosis but also cellular adhesion and intercellular liaison between the cells^57^. These results might identify one of the mechanisms by which TiO_2_ can enter the cell. In the neuronal system, 2 CHRNA genes, 1 after MPs and 1 after E171 exposure, were modulated and coding for cholinergic receptors. Cholinergic receptors are normally present in Caco-2 cells and after production of acetylcholine by the cells, a common effect in colorectal cells, the cholinergic receptor are affected and its dysregulation might be associated with inflammation and tumour development^58,59^.

Overall, the majority of the effects after exposure to E171 could be associated to the effects of the NPs and MPs fractions. However, some effects could only be attributed to the E171 itself. At the pathway level, metabolism of proteins with the insulin processing pathway and haemostasis were specific to E171 exposure. Effects in the expression of insulin genes were also observed *in vivo* by several groups^14,60^. At the GO term level, receptor activity, muscle adaptation, catalytic activity, organ development, and transcription factor were specific to E171 exposure. In addition, at the gene expression level, changes were observed in metabolism of RNA, chromatin organization, circadian clock, and programmed cell death. For these specific effects of E171, it should be taken into consideration that the NPs and MPs used do not fully cover the range of E171. The gene expression changes associated with oxidative stress support the detection of ROS formation in undifferentiated Caco-2 cells^8^ and *in vivo* transcriptomics analysis on colons of mice after oral administration of E171^14^.

Our study on ingestion of E171 in the mouse^14^ also found an effect on the mRNA levels in the colon related to oxidative stress, DNA repair, immune system, transport as well as signal transduction, neuronal system, and extracellular matrix organisation. In addition, the expression of seven orthologue genes affected after *in vivo* exposure were also found *in vitro* and were representative of the major effects of E171: immune system (BCL10, TNFAIP8L2, LRRC15), inflammation (LRRC15), signal transduction (TAS2R3), metabolism (BAAT, TNFAIP8L2, and BCL10), and cancer (WDR78). The WDR78 gene has been shown to be in the top 20 of hyper-methylated genes in CRC^61^, so the downregulation of WDR78 observed in our study could be caused by this. Furthermore, WDR78 is also significantly downregulated after exposure to TiO_2_ MPs, indicating that these MPs could also induce gene expression changes in relation to cancer.

To determine the relevance of our *in vitro* study for human exposure conditions, an estimation of human intake of TiO2 has been made^8^. According to several studies, humans are on average exposed to 1 mg/kg_bw_/day, implying that a 70 kg adult would ingest 70 mg of TiO_2_ per day. Assuming the production of 250 g faeces per day, the concentration of TiO_2_ would be 0.28 mg of TiO_2_/g of faeces. As faeces contain 75% of water, the density of faeces is approximately the same as water, thus the concentration of TiO_2_ would be 0.28 mg/mL of faeces. Translocation to other organs in the human body indicates a direct contact between the cellular wall of the intestines and E171^62^, Therefore, if we assume that only 1% is biologically available for exposure, 0.0028 mg/mL of TiO_2_ is potentially reaching the colon cells. The concentration used during the experiment is 0.01 mg/ml which is in the same order as the estimated concentration of TiO_2_ in the colon.

The present study provides an overview of the major effects on the transcriptome after exposure to E171 and the NPs and MPs fractions of TiO_2_. E171 causes the highest transcriptional impact both at the gene expression and pathway level, all pointing towards the facilitation of colon cancer development. Although both the NPs and MPs show lower but comparable levels of deregulated genes, biological interpretations showed that these fractions cause distinct cellular effects, So E171 shows a synergetic effect and is more than the added effect of the different size fractions. Taken together, these results indicate that the classification of E171 as an inert particle may need to be reconsidered and that NPs induce a more pronounced effect at the transcriptome level than MPs.Because a major part of our observations, like the immune responses, are still based on gene expression levels only, more mechanistically studies to support these observations are needed. Although changes at the level of gene expression or pathways cannot always be translated straightforward into changes at the level of proteins or specific markers e.g. the immune response. Based on our observations, we suggest performing a follow-up study focussing on phenotypical anchoring of the immune responses in which analyses a broad panel of cytokines and interleukins is included.

But since an increasing number of animal studies point out that E171 facilitate harmful effects in the colon including cancer development, the gene expression changes observed in this human colon cancer cell line delivers stronger evidence that exposure might also form an increased risk for humans. Although more evidence is needed from e.g. human intervention studies, suggestion by food industry and regulatory organisations to reduce the harmful effect by removing the NP fraction from E171, could be worthwhile.

## Materials and Methods

### Cell culture

The human colon carcinoma cell line Caco-2 (American Type Culture Collection ATCC, HTB-37) a frequently used colon cell line for *in vitro* toxicity studies, was cultured in an incubator under standard cell culture conditions (37 °C, 5% CO_2_) in Dulbecco’s Modified Eagles Medium (DMEM) (Sigma-Aldrich, the Netherlands). DMEM was supplemented with 10% FBS, 1% non-essential amino acids, 1% sodium pyruvate and 1% penicillin/streptomycin. Cells were passaged at 80 to 90% confluency.

### Characterisation of particles

E171 was kindly donated by Sensient Technologies, Mexico. TiO_2_ NPs were purchased at Io-Li-Tec, Germany, with 99.5% anatase particles. TiO_2_ MPs were customarily manufactured by PlasmaChem (Germany). Characterisation of the primary size of the particles was derived by SEM as well as a dynamic light scattering and zeta potential^8^. The similarity of the three-dimensional structures of E171, TiO_2_ NPs and MPs was confirmed by SEM. E171 contains slightly to fully rounded anatase particles with a proportion of 39% NPs and 61% MPs^8^. For NPs, the particle size ranged from 10 to 30 nm^8^, while the manufacturer indicated 15–25 nm, and a surface area of 50 to 150 m^2^/g. MPs had a crystal structure of anatase, a size higher than 100 nm and an average size of 622 nm^8^.

Dynamic light scattering results showed that the NPs and MPs had a zeta potential and a polydispersity index in the same range as E171. At a concentration of 1.10^−3^ mg/mL, E171 had the lowest hydrodynamic size in DMEM (316.8 ± 282.4 d.nm) compared to MPs (1385.83 ± 38.85 d.nm) and NPs the highest size (1942.17 ± 61.12 d.nm). The presence of serum reduced the agglomeration and aggregation of the particles^8^. Zeta potential results also showed that the addition of serum leads to more colloidal stability which indicates a decrease in the aggregation and agglomeration^8^. However, all results show that the suspension remained unstable.

### Particle exposure of Caco-2 cells

Caco-2 cells were used with a passage number ranging from 27 to 37. Each exposure was performed in triplicate and within each experiment, all the samples were in duplicate. Three days before exposure, ± 1.10^6^ Caco-2 cells were seeded in 21 cm^2^ culture dishes with 3 ml supplemented DMEM (Sigma Aldrich) and grown to reach 80 to 90% confluency.

E171 or TiO_2_ NPs or TiO_2_ MPs, were sterilised at 121 °C for 20 minutes prior to use, and subsequently dispersed in glass tubes at a concentration of 1 mg/ml in supplemented DMEM where FBS was replaced by 0.05% BSA in order to reduce the antioxidant effect of FBS. The solutions were vortexed and subsequently sonicated for 30 minutes at 40 KHz in a bath sonicator (Branson 2200). After sonication, the particles were further dissolved to 0.01 mg/ml in 0.05% BSA supplemented DMEM. Prior to exposure, cells were washed twice with Hank’s Balanced Salt Solution (HBSS) without Ca^2+^and Mg^2+^ (Sigma-Aldrich, The Netherlands). Cells were exposed to 3 ml of 0.01 mg/ml TiO_2_ NPs, MPs or E171 for 2, 4 or 24 hours which gives a final concentration of 1.43 µg/cm^2^. This concentration was previously shown by our group to be the lowest non-cytotoxic^8^. Timepoints were based on a previous study^63^ that showed internalisation of TiO2 NPs after 2 h and reached saturation after 8 h, so that in our study we investigated early as well as prolonged effects of internalisation. After exposure, cells were washed with HBSS (without Ca^2+^and Mg^2+^) once and lysed using 1 ml Qiazol Lysis Reagent (Qiagen, The Netherlands). The Qiazol cell solution was stored at −80 °C until RNA isolation.

### RNA isolation

Total RNA was isolated from undifferentiated Caco-2 cells exposed to TiO_2_ NPs, TiO_2_ MPs, and E171 in Qiazol (Qiagen, The Netherlands) using the miRNeasy Mini Kit (Qiagen, The Netherlands) according to the manufacturer’s protocols for Animal Cells and Animal Tissues including a DNase treatment^64^. Total RNA yield as well as the 260/230 and the 260/280 were measured on a Nanodrop® ND-1000 spectrophotometer (Thermo Fischer, The Netherlands). Samples with a 260/230 ratio between 1.8 and 2.0 and a 260/280 ratio between 1.9 and 2.1 were checked for their integrity. The integrity of total RNA was checked using RNA Nanochips on a 2100 Bioanalyzer (Agilent Technologies, The Netherlands). All samples had on average a RNA integrity number (RIN) of 9.5 ± 0.4, which is above 6, the number at which each sample is approved for microarray analysis.

### cRNA synthesis, labelling and hybridization

Total RNA was converted into cRNA and labelled according to the One-Color Microarray-Based Gene Expression Analysis protocol version 6.6 (Agilent Technologies, The Netherlands)^65^ as described previously^66^. In brief, 200 ng of total RNA was diluted in Spike Mix. The primer and the template were denaturated by using a thermocycler (Tprofessional, Biometra, Germany) at 65 °C for 15 minutes. cDNA was synthetized by incubating each sample with cDNA Master Mix in a thermocycler at 40 °C for two hours and 70 °C for 15 minutes. Afterwards, cRNA was synthetized and labelled by incubating Transcription Master Mix, containing the cyanine 3-CTP dye, with the cDNA at 40 °C on a thermocycler for two hours. The RNeasy Mini Kit was used to purify the amplified cRNA samples according to the manufacturer’s protocol of Agilent. Subsequently, the cRNA was quantified using a spectrophotometer with the Microarray Measurement function. The yield and specific activity of Cy3 bound to the cRNA was determined using the formulas in the manufacturer’s protocol of Agilent.

After incubating cRNA samples with Gene Expression Blocking Agent and Fragmentation Buffer (Agilent Technologies) at 60 °C for 30 minutes, hybridization was performed on SurePrint G3 Human GE 8 × 60k V2 slides and a hybridization oven (Shel Lab, United States) was used at 65 °C for 17 hours. After hybridization, the microarray slides were washed with Gene Expression wash buffers (Agilent Technologies, The Netherlands) and scanned using an Agilent DNA Microarray Scanner with Surescan High-resolution Technology (Agilent Technologies, The Netherlands) with scanner settings to Dye Channel: G, Profile: AgilentG3_GX_1Color, Scan region: Agilent HD (61 × 21.3 mm), Scan resolution 3 µm, Tiff file dynamic range: 20 bit, Red PMT gain: 100%, and Green PMT gain: 100%.

### Pre-processing and data analysis of microarrays

Pre-processing methods used were described previously^14^. In short, all samples met the quality criteria of the Feature extraction software (FES) (version 10.7.3.1) from Agilent. An in-house QC pipeline was developed and published (github.com/BiGCAT-UM/arrayQC_Module) to thoroughly check the quality and normalize the data as follows: local background correction, flagging of bad spots, controls and spots without adequate intensity, log2 transformation and quantile normalization. Five samples out of 71 did not pass the quality control the first time. These samples were re-run and were validated by the QC the second time. Raw data that has both expression values and genes were selected for data analysis based on flags and missing values (GEO accession: GSE110410).

First pre-processing was performed with the samples of the time course experiment with NPs. For this, six groups were defined: NPs 2 h, 4 h, and 24 h for the exposed samples and control 2 h, 4 h, and 24 h for the controls. The second pre-processing was split in 2 parts in which the first part was a pre-processing of the NPs 24 h with its time-matched control and the second part was a pre-processing of E171 24 h and MPs 24 h with their time-matched control. The second pre-processing was split in 2 because the experiments were performed independently.

After defining the different groups for the pre-processing, the next steps were exactly the same for all groups. Within each group, probes were flagged for bad spots and at least 30% of all samples had to have good spots. Spot identifiers were deleted when more than 40% of samples in each group had a missing value. Background correction was performed by removing the spot identifiers that had an average expression less than four in all groups. Missing values were imputed by k-nearest neighbours using the standard settings of the GenePattern ImputeMissingValues.KNN module v13^67^. Spot identifiers were annotated to Agilent probe identifiers and merging of exact same probe identifiers with the median method for pre-processing data was performed in Babelomics 5^68^. Next, Agilent probe identifiers were re-annotated to EntrezGeneIDs. Merging of the expression data for all genes with an identical EntrezGeneIDs was done with Babelomics 5 using the median method for pre-processing data.

Differentially expressed genes (DEG) were identified by using a Linear Mixed Model Analysis for Microarrays (LIMMA) (version 1.0) R-script with cut-off values of a fold-change (FC) of 1.2, 1.5, and 1.8 and a p-value of 0.05^69^. The data was paired for replicates. Furthermore, according to the Benjamini-Hochberg method with a threshold at 0.05, the false discovery rate was calculated. With the LIMMA analysis, the number of DEG were identified from each sample; data of the exposed samples were corrected to the data from its time matched control sample.

### Pathway analyses

Over-representation gene set analysis (ORA) was performed with Consensus Pathway Database (CPDB) with the DEG of each time point^70^. CPDB is an aggregate of 16 different databases developed by the Max Planck institute and its functionalities have been explained in Nature Protocols^71^. CPDB has a focus on molecular interactions, and identifies pathways associated with DEG. For computing the significance of the over-representation of the annotation sets with respect to user-input molecules, CPDB applies Fisher’s exact test. For each annotation set, a p-value is calculated. As many annotation sets are tested, CPBD corrects for multiple hypothesis testing using the false discovery rate procedure within each type of annotation set^70,71^. All the available databases from CPDB were used (release 32, 1 Nov. 2017) with settings in the “pathways as defined by pathway databases” with a minimum overlap of input list of 2 and a p-value cut-off of p < 0.01. As shown previously, the pathway analysis is limited to the number of genes that can be mapped to a pathway which is often less than 50% of the input list^14^. In order to retrieve the maximal amount of relevant biological information from our gene lists, the corresponding GO terms were also determined.

### Gene Ontology (GO) term classification

GO categories related to the DEG were identified by CPBD. In order to retrieve more biological information, GO terms of level 2, 3, 4, and 5 were selected with a p-value cut off of 0.01. Biological processes of each GO term were retrieved based on GO terminology.
